# Family Nurses' Experience With and Fidelity to a Family Support Intervention in Intensive Care Units: A Mixed‐Methods Study

**DOI:** 10.1002/nop2.70908

**Published:** 2026-11-13

**Authors:** Lotte Verweij, Saskia Oesch, Marco Riguzzi, Simone Sutter, Rahel Naef

**Affiliations:** ^1^ Faculty of Medicine, Implementation Science in Nursing University of Zurich Zurich Switzerland; ^2^ Center for Clinical Nursing Science University Hospital Zurich Zurich Switzerland

**Keywords:** complex health intervention research, critical care, family nursing, fidelity, implementation science

## Abstract

**Aim:**

To assess family nurses' fidelity – comprehension (*receipt*) and performance (*enactment*) and experiences with delivering a family support intervention.

**Design:**

An embedded longitudinal convergent mixed‐methods study.

**Review Methods:**

*Receipt* was assessed using the Family Nursing Practice Scale and case‐conference attendance. *Enactment* through an intervention‐performance self‐rating and observation tool, and family nurses' availability and capacity measures for intervention delivery. Two focus groups explored nurses' experiences. Quantitative and qualitative data were analysed using non‐parametric tests and inductive content analysis and thereafter integrated in line with the contiguous approach.

**Data Sources:**

Data were collected among all 22 family nurses from eight adult intensive care units between April 2022–January 2024: pre‐, mid‐ (3–9 months) and post‐implementation (18–24 months).

**Results:**

Fidelity *receipt* was moderate to high and *enactment* was consistently high, particularly in relational and supportive activities. Fidelity was shaped by three concepts: role development, enactment and support, underscoring that effective intervention delivery requires both skills and a supportive environment.

**Conclusion:**

High fidelity of nurse‐led family interventions is feasible when ongoing mentorship, protected time, clear role definitions and interprofessional support are considered.

**Implications for the Profession:**

Critical care nurses can develop skills and competencies to deliver specialized family care. Support to develop nurses' practical, relational and interprofessional skills and emotional resilience should be considered as well as leadership support and team acceptance.

**Impact:**

- A family nursing role within intensive care enables delivery of family‐focused care and requires practical, relational and interprofessional competencies to support high‐fidelity delivery.
- Role definitions and interprofessional team acceptance facilitate intervention enactment and support the integration of family‐focused care.
- Reflection and leadership support are essential to develop family‐focused care skills.

**Reporting Method:**

GRAMMS checklist for mixed‐methods evaluations.

**Patient or Public Contribution:**

A patient and family advisory board was closely involved throughout the research process. The board helped to ensure that research remained relevant and aligned with the needs and perspectives of the target population.

## 1 Introduction

Family members of critically ill or severely injured patients are often deeply affected by the uncertainty and stressful situation (Saeid et al. 2020; Shirasaki et al. 2024; Smith et al. 2025). They experience significant distress and suffering, with up to two thirds of family members being affected by long‐term (or post‐ICU) symptoms such as anxiety, depression, post‐traumatic stress disorder, collectively referred to as Post‐Intensive Care Syndrome‐Family (PICS‐F), which may also negatively affect family functioning. Moreover, not attending to families' needs may further exacerbate their suffering and increase the risks of PICS‐F (Azoulay et al. 2025).

Family‐focused interventions, defined as interventions that involve family members and/or patients and target cognitive, affective and behavioural levels (Danford et al. 2024), have shown potential benefits for PICS‐F, but findings remain contradictory (Coventry et al. 2023; Duong et al. 2024; Kiwanuka et al. 2022; Lebel and Charette 2021; Rhoads et al. 2022; Xyrichis et al. 2021). Interventions include, among others, psychoeducational, relationship‐focused, behaviour‐changing interventions aimed at strengthening families, alleviating their suffering and improving their health and well‐being, quality of life and family functioning (Bohart et al. 2024; Coventry et al. 2023; Kiwanuka et al. 2022). One such intervention, the FICUS family support intervention (FSI), was implemented as part of the FICUS trial (Naef et al. 2025; Riguzzi et al. 2026). The FSI was grounded in a family systems nursing approach (Bell and Wright 2021; Shajani and Snell 2023) and was evaluated for its ability to improve quality of communication and support, reduce family burden and distress, and enhance family health and well‐being (Naef et al. 2022, 2025).

The FSI includes three interrelated core components delivered to families by critical care nurses who take on the role of a family nurse (FN); (1) *engaging and liaising*, including relationship building with families, connecting and coordinating family care activities, and transition and follow‐up care; (2) *supporting*, including therapeutic nurse‐family conversations and psychoeducational and relationship‐focused activities; and (3) *communicating*, including structured interprofessional family meetings and shared decision‐making with families. The FSI was considered a complex intervention because of its interrelated and adaptive components, which were delivered by FNs within the highly complex and dynamic interprofessional ICU environment (Skivington, Craig, et al. 2024; Skivington, Matthews, et al. 2024). The main results of the FICUS trial showed beneficial effects of the FSI on family satisfaction with ICU care, quality of communication, and on cognitive and emotional support, but did not yield significant or conclusive results regarding PICS‐F or family functioning (Riguzzi et al. 2026).

Family‐focused care is an important component of critical care nursing, and nurses are well positioned to establish relationships with families, identify and respond to their needs, and facilitate communication and shared decision‐making throughout the ICU trajectory (Hwang et al. 2025). However, delivering family‐focused interventions in routine practice requires nurses to integrate new knowledge and skills into complex and rapidly changing clinical environments. Understanding how nurses learn, interpret and apply such interventions is therefore important not only for evaluating intervention fidelity, but also for strengthening nurses' capacity to provide effective family care in critical care practice (Oesch, Verweij, Riguzzi, Finch, Clack, and Naef 2025; Oesch, Verweij, Riguzzi, Finch, and Naef 2025).

Evaluating how critical care nurses, in a novel role as a FN, enact a complex family support intervention such as the FSI may provide additional insights into the FICUS trial outcomes and its implementability, while also informing the education, support and implementation of FSIs in critical care practice.

## 2 Background

Fidelity to a manualized intervention, such as the FSI is a central process indicator in complex health intervention research and a proximal implementation outcome (Proctor et al. 2023). It has traditionally been defined as the degree to which an intervention is delivered as manualized (Dusenbury et al. 2003; Proctor et al. 2011, 2023). However, fidelity is a multi‐dimensional outcome, particularly in complex health interventions, where the intervention's effect depends not only on what is delivered but also on how those delivering it understand and enact the intervention (Bellg Borrelli et al. 2004; Richards and Hallberg 2015; Schultes et al. 2021; Zvoch 2012).

To address the need for a clear conceptualization of intervention fidelity, Bellg Borrelli et al. (2004) defined fidelity dimensions in addition to the traditionally known intervention *delivery*. Fidelity *receipt* was defined as the degree to which deliverers comprehend and internalize intervention content, whereas fidelity *enactment* was defined as the extent to which learned skills and behaviours are applied in real‐world contexts (Bellg Borrelli et al. 2004). Assessing these fidelity dimensions individually enables researchers to more accurately interpret outcomes of intervention effectiveness. For instance, distinguishing between implementation failure, defined as the inability to deliver the intervention as manualized, often due to issues of adherence, intervention comprehension or available resources, and limitations inherent to the intervention itself is important when interpreting intervention outcomes (Albers et al. 2024; Fixsen 2025; Gearing et al. 2011; Ginsburg et al. 2021; MacEachern et al. 2023; Skivington, Craig, et al. 2024).

Assessment of fidelity is particularly challenging in dynamic healthcare contexts such as ICUs, where interventions must be tailored to patients' clinical trajectories, family needs and interprofessional team processes (Paez et al. 2024; Páez et al. 2025). For nurses taking on new or expanded roles, fidelity may further depend on their ability to acquire and translate intervention‐specific knowledge and skills into clinical practice. Evaluating the fidelity of FNs beyond the delivery of the FSI and focusing on how they learn (receipt) and enact (perform) the FSI and translate their skills into practice may therefore provide valuable insight into how complex FSIs can be effectively integrated into critical care nursing practice. This may also inform the education and support required for nurses to adopt and sustain new family‐nursing roles.

## 3 The Study

### 3.1 Aims and Objectives

The aim of this study was to evaluate how FNs comprehended (fidelity *receipt*) and performed (fidelity *enactment*) the FSI within the context of the FICUS trial and in addition to evaluate FNs' experience with delivering the novel FSI and their role within the interprofessional ICU team.

## 4 Methods

### 4.1 Design

A longitudinal, convergent mixed‐methods evaluation (Creswell 2021; Fetters et al. 2013) which was part of a larger implementation study (Oesch et al. 2023) that was carried out parallel to the FICUS cluster‐randomized clinical trial (Naef et al. 2022, 2025), was conducted between April 2022 and January 2024. Data were collected through questionnaires, observations and focus group interviews at three time points; before FSI implementation (*pre*); during the active implementation phase, that is, 3–9 months after implementation start (*mid*), and at the end of the active implementation phase, that is, 18–24 months after implementation start (*post*), see Table 1. The manuscript followed the GRAMMS checklist for mixed‐methods evaluations (O'Cathain et al. 2008) (see Supplementary File S1).

**TABLE 1:** Operationalization of fidelity receipt and enactment.

Fidelity domain	Operationalization fidelity categories	Measure	Scores	Source	Timepoint^a^		
					Pre	Mid	Post
**Receipt**							
Case conference attendance	FNs' attendance rate during monthly case conferences: low (0%–50%), moderate (51%–79%), high (80%–100%) (*n*, %)	Structured case conference attendance form for FNs	—	Structured intervention log		X	X
Skills	FNs' appraisal of own skills: low (0%–50% of scale range), moderate (51%–79%), high (80%–100%) (*n*, %)	German Family Nursing Practice Scale (Naef et al. 2021)	1.0–5.0 (low), (inverse score)	Questionnaire	X	X	X
**Enactment**							
Performance	Quality and consistency of FSI performance: low (0%–50% of scale range), moderate (51%–79%), high (80%–100%) (*n*, %)	FSI self‐rating and observation Tool (Oesch et al. 2023). Items per FSI component: 16 engaging and liaising; 28 supporting; 16 communicating.	Self‐rating 5‐point Likert‐scale, range: 60–300. Component ranges: 16–80 28–140 16–80	Questionnaire		X	X
			Observation 3‐point Likert‐scale. Component ranges: 16–48 28–84 16–48 (inverse score)	Observation		X	
Availability and capacity	FNs' availability and capacity for FSI delivery, categorized as low: FN is never or rarely available or capable; moderate: FN is sometimes or often available or capable; high: FN is always available or capable (*n*, %)	FSI capacity based on two statements: In the ICU, one of the two FNs was available 5 days a week to provide the FSI. I was able to provide the FSI to families as specified in the manual and based on the family's needs.	5‐point Likert‐scale. Score range 1–5 per question.	Questionnaire		X	
**Receipt and enactment**							
Comprehension and performance	Experiences in working systemically with families.	Focus group interviews.	—	Interview			X

*Note:* This table has been adapted from the original published in Albers et al. (2024), Implementation Science https://doi.org/10.1186/s13012‐024‐01406‐3.

Abbreviations: FN, family nurse; FSI, family support intervention; ICU, intensive care unit.

^a^ Timepoint assessment, Pre = before FSI implementation, Mid = 3–9 months after FSI start, Post = 18–24 months after FSI start.

### 4.2 Theoretical Framework

We conceptualized intervention fidelity using an adapted version of Carrol and colleagues' Conceptual Framework for Implementation Fidelity (2007) combined with the fidelity domains outlined by Bellg Borrelli et al. (2004), see Figure 1. Fidelity *receipt* refers to the degree to which FNs comprehended the FSI, including both cognitive and behavioural skills. Fidelity *enactment* was defined as FNs' engagement in and performance of FSI core activities (Albers et al. 2024; Oesch et al. 2023). This study focused on the evaluation of fidelity *receipt* and *enactment*, while evaluation of fidelity *adherence* (*delivery*) is reported elsewhere (Naef et al. 2025).

**FIGURE 1:**
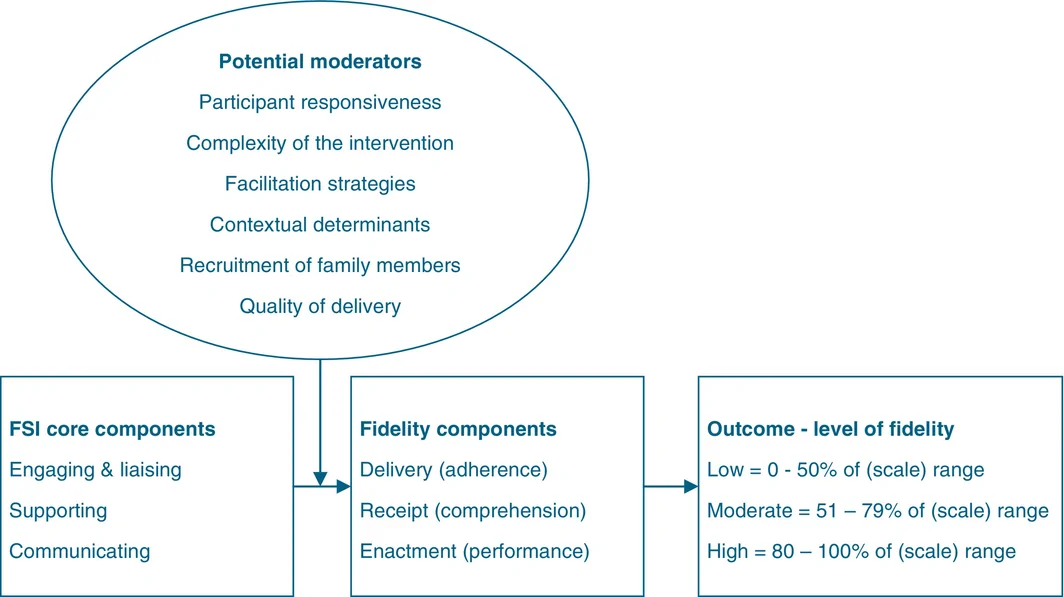
Conceptual framework for FSI fidelity. This framework has been adapted from the original framework by Carroll et al. (2007), Implementation Science https://doi.org/10.1186/1748‐5908‐2‐40 and has previously been published in Albers et al. (2024), Implementation Science https://doi.org/10.1186/s13012‐024‐01406‐3. The figure is licensed under a Creative Commons Attribution 4.0 International Licence (CC BY 4.0 OA). FSI, Family Support Intervention.

### 4.3 The Family Support Intervention and Its Implementation

The FSI consisted of a care pathway including three core components: (1) engaging and liaising, (2) supporting and (3) communicating, which were delivered by FNs to families during patients' ICU stay and thereafter, see Figure 2 (Naef et al. 2022). FNs' core responsibilities included conducting regular meetings with families to assess family needs in the burdensome situation, provide relationship‐focused and psychoeducational support, and organization and facilitation of interprofessional family meetings to promote effective communication and shared decision‐making. The core components were standardized, and the minimal intervention dose was defined, but FNs were instructed to adapt the dose and frequency of each intervention component based on their clinical judgement to individual families' needs and to the course of illness during patients' ICU stay and up to 3 months thereafter. Further details on the FSI are reported in the FICUS study protocol (Naef et al. 2022).

**FIGURE 2:**
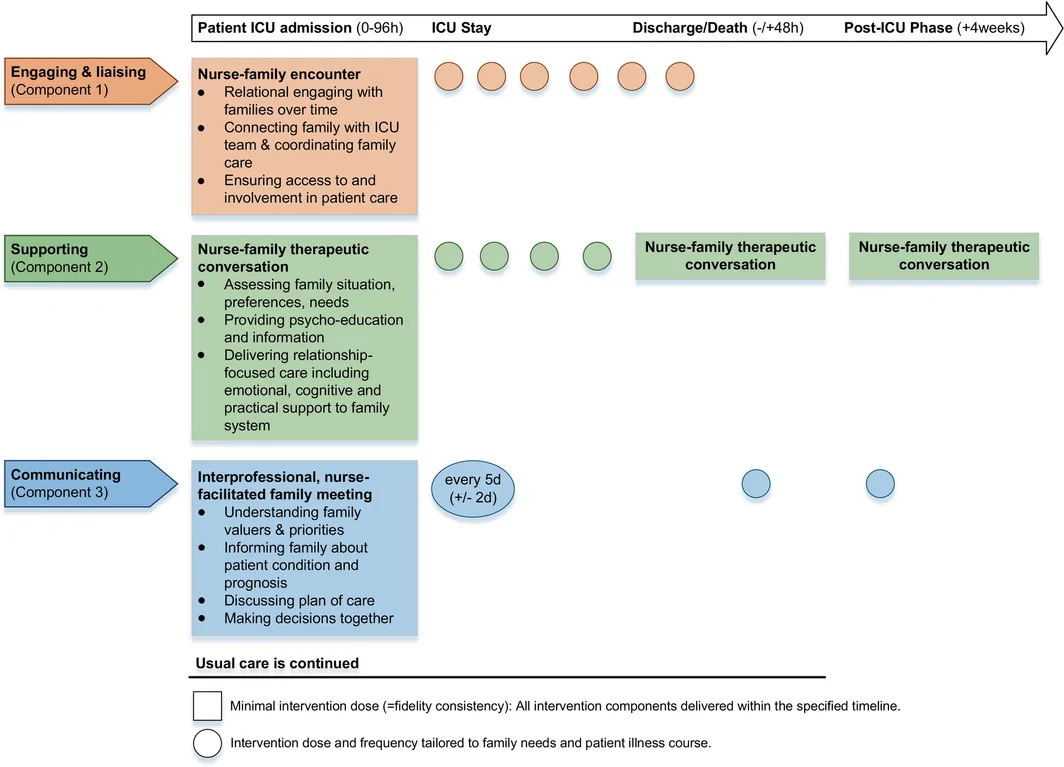
Family support intervention. This figure has previously been published by Naef et al. (2025) *JAMA*, https://doi.org/10.1001/jamainternmed.2025.3406 and is licensed under a Creative Commons Attribution 4.0 International Licence (CC BY 4.0 OA). d, days; h, hours.

Each ICU appointed at least two FNs responsible for the FSI delivery and for which they received protected time outside of their shiftwork. All FNs completed a five‐day training programme delivered by the research team and an external expert in family systems care before implementation start. Implementation support was provided to each ICU by a tailored and adaptive implementation approach including leadership support, a continuous learning collaborative for family nurses (case conferences, refresher days), clinical support and interactive external assistance. Local implementation teams were formed to support the FSI implementation and included the FNs, an implementation support person, and nurse and physician leaders, locally responsible for the implementation (Naef et al. 2022, 2025; Oesch et al. 2023).

### 4.4 Sampling and Recruitment

This study was conducted in eight ICUs in the German‐speaking part of Switzerland, which were randomized to the intervention arms of the FICUS trial. ICUs operated a median of 11 beds (range 9–20), with a median patient length of stay of 4 days (range 2–5) per admission. On average, ICU nursing teams consisted of 56 ICU nurses. Participants were all certified critical care nurses who took on the role of FN in each intervention ICU.

### 4.5 Population and Sample

All 22 FNs who were involved in FSI delivery in the context of the FICUS Trial, participated in the study. Over the course of the trial, some FNs left the ICU, resulting in lower participant rates at timepoints *mid* and *post*.

### 4.6 Exclusion Criteria

No exclusion criteria were applied.

### 4.7 Data Abstraction and Sources

#### 4.7.1 Fidelity Receipt

Fidelity *receipt* was operationalized as FNs' attendance during monthly case conferences to support FSI comprehension and practices as well as family nursing skills as assessed by the psychometrically validated German version of the Family Nursing Practice Scale (FNPS) (Naef et al. 2021; Simpson and Tarrant 2006), see Table 1.

##### 4.7.1.1 Family Nurses' Case Conference Attendance

FNs' online case conference attendance was registered by a designated member of the research team during the active trial phase on a standardized registration form.

##### 4.7.1.2 Family Nurses' Skills

The 10‐item German Family Nursing Practice Scale (FNPS) was assessed at timepoints *pre, mid* and *post* by the FNs from each ICU. Questions were answered on a 5‐point Likert scale with lower scores indicating higher proficiency (Naef et al. 2021; Simpson and Tarrant 2006). The German FNPS demonstrated high internal consistency in the validation study (*α* = 0.84) (Naef et al. 2021) which was confirmed in the current sample with Cronbach's *α* of 0.86 at *pre*, 0.80 at *mid* and 0.86 at *post*.

#### 4.7.2 Fidelity Enactment

Fidelity *enactment* was operationalized as FNs' FSI performance and measured using a specifically developed, FSI‐manual‐based 60‐item self‐rating and observation tool (Oesch et al. 2023), see Supplementary File S2. These tools include operationalized items of each intervention activity of the manualized FSI components. The self‐rating tool aimed to obtain information on FNs' personal view on their FSI performance, based on the last 3–5 families they had cared for, whereas the observation tool served the structured observation of the intervention performance in one specific family nurse‐family interaction. In addition, FNs' availability and capacity were assessed by two self‐developed questions, see Table 1.

##### 4.7.2.1 FSI Self‐Assessment

Self‐assessment took place at timepoints *mid* and *post* and used a 5‐point Likert scale to let FNs individually indicate a differentiated level of FSI activity performance (never, rarely, sometimes, often, always) (Oesch et al. 2023). The total score ranged between 60 and 300, with a lower score indicating a lower level of performance. In addition, each FSI component was rated separately and included score ranges between 16 and 80 for component 1 – *engaging & liaising* and 3 – *communicating* and 28–140 for component 2 – *supporting*. The self‐assessment was pre‐tested by two FNs who evaluated the instrument's content and level of assessment. Based on their feedback, the instrument was adjusted and finalized.

##### 4.7.2.2 FSI Observation

Per ICU, one of the FNs (*n* = 8) was observed once, during timepoint *mid*, by one of the two observers during performance of at least one FSI component in the context of a nurse‐family interaction. The FSI activities were assessed on a 3‐point scale to indicate if activities were performed, partly performed or not performed, scores ranges: 16–48 for component 1 and 3, and 28–84 for component 2. Lower scores indicated higher performance (Oesch et al. 2023). Observations were performed only once per ICU and by one observer because of the impact on privacy during the nurse‐family interactions. Therefore, no inter‐rater reliability measure could be calculated. Observations were performed by a doctoral and a senior researcher, both trained in family system nursing and qualitative research. The instrument was pre‐tested once by one researcher. To avoid discrepancies in observations, the observers jointly discussed the tool, the interpretation of the observation items and the scoring system. In addition, after each observation, the observers discussed findings to calibrate following observations and interpretation.

##### 4.7.2.3 FN Availability and Capacity for FSI Delivery

To evaluate FN availability and capacity to provide family support, two self‐developed statements were administered to local ICU study teams at timepoint *mid*: referring to the last 4 weeks, (1) ‘In the ICU, one of the two FNs was available five days a week to provide the FSI’, and to FNs only (2) ‘I was able to provide the FSI to participants as required in terms of time, based on the protocol and the family's needs’. Answers were provided on a 5‐point Likert scale to provide a differentiated view on their availability and capacity to provide the FSI (never, rarely, sometimes, often, always) (Oesch et al. 2023).

#### 4.7.3 Family Nurses' Experiences (Fidelity Receipt and Enactment)

In addition to the above‐mentioned assessment of fidelity *receipt and enactment*, FNs' experiences with the FSI were evaluated through two semi‐structured focus group interviews at the end of the active implementation phase (*post*). The aim was to explore (1) how FNs developed and comprehended skills in family systems care and the FSI components, and (2) how FNs enacted their role within the interprofessional ICU teams. The interview guide was structured around key themes such as (1) strategies supporting FN skill development, (2) role performance within the interprofessional ICU context, and (3) experiences with FN role delivery within the ICU nursing teams, see Supplementary File S3.

The first focus group interview took place in October 2023 with FNs from four ICUs and the second interview took place in January 2024 with FNs from the remaining four ICUs. Interviews were held in person at a central location, chosen for their accessibility for most participants. Both interviews were moderated by a doctoral student who was a research team member [S.O.] and co‐moderated by an experienced external researcher. Each interview lasted approximately 120 min and was audio‐recorded.

#### 4.7.4 Participant Characteristics

Characteristics of participants were collected during timepoint *pre*, *mid* and *post* and included age, sex, level of education, years of ICU work experience, prior training in family systems care, and full‐time equivalent employment in the ICU.

### 4.8 Data Collection

FNs were invited to fill out questionnaires by a face‐to‐face approach during their attendance at the FSI training activities, during onsite visits from the research team and after the focus group interview. When the data‐collection timepoints did not align with onsite activities, FNs were approached by a member of the research team by e‐mail. Reminder e‐mails were sent after 2 weeks. By filling out and returning the questionnaire, informed consent was provided.

For onsite observations, FNs were contacted by a member of the research team to schedule an observation of an FSI activity. FNs were verbally informed about the purpose of the observations and asked to sign an informed consent form for the use of pseudonymized data prior to the observation.

To participate in the focus group interviews, FNs were invited via email by a member of the research team. FNs received detailed written information about the aim, interview procedures, and an informed consent form and were offered the opportunity to ask questions before agreeing on participation. Written informed consent was collected immediately prior to each interview.

### 4.9 Data Analysis

All qualitatively collected data were pseudonymized prior to analysis, and questionnaires and observation forms were coded to remove identifying information. All data were stored on secured university servers only accessible by members of the research team.

#### 4.9.1 Quantitative Data Analysis

Data were analysed using IBM SPSS Statistics (Version 30.0) and summarized with descriptive statistics, including medians with ranges and frequencies and percentages. Wilson's confidence interval was computed for proportions. The exact confidence interval of medians was calculated based on the binomial distribution. Comparisons between timepoints were conducted using the Hodges–Lehmann location shift estimate of the paired median difference, the non‐parametric Friedman test (more than two timepoints), and the Wilcoxon Signed‐Rank test (with Holm‐Bonferroni correction for pairwise comparisons *pre* & *mid, pre* & *post* and *mid* & *post*).

Given a small proportion of missing data and the small sample size, complete‐case analyses were conducted. Cases with missing values for variables required for a given analysis were excluded from that analysis, and no data imputation was performed.

#### 4.9.2 Level of Fidelity

The level of fidelity was pre‐defined in categories of *low, moderate* and *high* for each measure of fidelity *receipt* and *enactment* referring to 0%–50%, 51%–79% and 80%–100% of (scale) range, respectively (Albers et al. 2024) and reported as *n* (%) of FNs for each fidelity category. The definition of fidelity was based on the theorized definition used by Borrelli et al. (2005) and Garbacz et al. (2014).

#### 4.9.3 Qualitative Data Analysis

Audio‐recorded interviews were transcribed verbatim and verified for accuracy by the second author. Data were analysed using MAXQDA (2024) following an inductive content analysis approach (Erlingsson and Brysiewicz 2017; Graneheim et al. 2017) to identify themes and patterns across the data (Elo and Kyngäs 2008). The analysis process started by reading the transcripts entirely to gain familiarity with the data. Meaning units, quotes relevant to the research questions, were identified, condensed, and inductively coded. Codes were then refined into subcategories and categories, capturing both manifest and latent meanings. Preliminary categories were further developed, grouped and abstracted into overarching themes regarding FN cognitive and behavioural skill development, engagement and performance of the FSI. Quotations were used to illustrate each theme. Data analysis was conducted by the first [L.V.] and second [S.O.] author and discussed in regular meetings with the last author [R.N.] to refine coding and ensure consensus.

#### 4.9.4 Data Integration

Data integration was conducted using a contiguous approach (Fetters et al. 2013). Both quantitative and qualitative data were initially analysed independently. Subsequently, the findings were brought together and systematically compared by L.V. and S.O. using a joint display. Quantitative results were aligned with the corresponding qualitative themes to identify areas of convergence and discrepancies. Where findings converged, the consistency of the evidence across data types was considered; where findings differed, discrepancies were examined and discussed in relation to the substantive meaning and context of the findings. The interpretation of the integrated findings was subsequently discussed with the last author [R.N.] to reach consensus. Based on this integrative process, meta‐inferences were developed that reflected conclusions arising from the combined quantitative and qualitative evidence. In line with the contiguous approach, the integrated findings and resulting meta‐inferences are presented and further interpreted in the discussion section.

### 4.10 Ethical Considerations

The FICUS trial was approved by the responsible Swiss cantonal ethics committee (No. 2021‐02300). For this embedded mixed‐methods study, the lead ethics committee waived the need for additional ethical approval (BASEC‐Nr. Req‐2023‐01026). We followed the national guideline of research with humans (SAMS 2015).

### 4.11 Rigour

Several strategies were applied to ensure rigour and trustworthiness in the qualitative analysis (Graneheim et al. 2017). Data were collected by a doctoral [S.O.] and senior researcher, trained in qualitative research who was external to the research project. The analysis was conducted by three researchers [L.V., S.O., R.N.] to minimize single‐perspective bias and to resolve divergent interpretations through consensus (Carter et al. 2014). Contextualization of themes was verified through discussion and cross‐checking with participant quotations (Creswell and Plano Clark 2018).

## 5 Findings

### 5.1 Participants

A total of 22 critical care FNs participated in the study. All 22 completed baseline data collection, while 17 or 18 completed the data collection at timepoint *mid* and 15, 18 or 22 completed data collection at timepoint *post*, depending on the reported outcome. FNs' demographic characteristics for each timepoint are presented in Table 2. At *post*‐implementation, seven FNs participated in the first focus group interview and 11 in the second. These were the same participants who completed the questionnaire at timepoint *post*.

**TABLE 2:** Participant characteristics.

Demographics/data collection timepoint	Pre^a^	Mid^a^	Post^a^
	*n* = 22	*n* = 18	*n* = 18
Age (years), median (min, max)	41 (26, 57)	41 (26, 57)	40.5 (27, 58)
Sex, *n* (%)			
Female	20 (91)	17 (94)	17 (94)
Male	2 (9)	1 (6)	1 (6)
Highest level of education, *n* (%)			
Diploma degree in Nursing	14 (64)	12 (63)	10 (55)
Bachelor of Science in Nursing	6 (27)	4 (22)	7 (39)
Master of Science in Nursing	2 (9)	2 (11)	1 (6)
Total work experience (years), median (min, max)	19 (6, 36)	20 (6, 36)	21.5 (4, 37)
ICU work experience (years), median (min, max)	9.5 (3, 29)	9.5 (3, 29)	12 (3, 31)
Training in family systems nursing,^b^ *n* (%)			
Yes	8 (36)	6 (33)	7 (38.8)
No	14 (64)	12 (67)	11 (61)
Full‐time equivalent, median (min, max)	90 (60,100)	90 (60, 100)	90 (70, 100)

Abbreviation: ICU, intensive care unit.

^a^ Pre = before Family Support Intervention (FSI) implementation, Mid = 3–9 months after FSI start, Post = 18–24 months after FSI start.

^b^ Before study start: Certificate of Advanced Studies in Family Care, Master of Advanced Studies in Patient and Family education, Family Systems Care modules within the bachelor's or master's programme.

### 5.2 Fidelity Receipt

#### 5.2.1 Family Nurses' Case Conference Attendance

The overall attendance rate was 63.5% (95% confidence interval (CI) 43.0%–80.3%) over 14 conferences among 22 FNs, see Table 3. In total, 7 (31.8%) FNs attended ≤ 50% of the case conferences, 11 (50%) between 51%–79% and 4 (18.2%) attended 80%–100% of the conferences, referring to low, moderate and high‐fidelity levels, respectively.

**TABLE 3:** Family nurse fidelity receipt and enactment.

Level of fidelity receipt	Pre^d^	Mid^d^	Post^d^	Difference	*p*
FN case conference attendance			*n* = 22		
Overall attendance rate during 14 case conferences, % [95% CI]^e^	—	—	63.5 [43.0–80.3]	—	—
Low fidelity (0%–50%), *n* (%)	—	—	7 (31.8)	—	—
Moderate fidelity (51%–79%), *n* (%)	—	—	11 (50.0)	—	—
High fidelity (80%–100%), *n* (%)	—	—	4 (18.2)	—	—
FNPS^a^ sum score (inverse)	*n* = 22	*n* = 17	*n* = 15		
Median [95% CI]^f^	2.0 [1.7–2.4]	1.7 [1.4–2.0]	1.6 [1.3–2.0]	—	<.001^h^
Min, Max	1.2, 3.6	1.1, 2.3	1.0, 2.9		
Comparison pre‐mid (*n* = 17)^g^	—	—	—	−0.4 [−0.7 to −0.2]	0.006^i^
Comparison pre‐post (*n* = 15)^g^	—	—	—	−0.5 [−0.8 to −0.2]	0.012^i^
Comparison mid‐post (*n* = 15)^g^	—	—	—	−0.1 [−0.4–0]	0.304^i^
Low fidelity (range > 3.0), *n* (%)	2 (9.1)	0 (0)	0 (0)	—	—
Moderate fidelity (range1.9–3.0), *n* (%)	13 (59.1)	7 (41.2)	5 (33.3)	—	—
High fidelity (range ≤ 1.8), *n* (%)	7 (31.8)	10 (58.8)	10 (66.6)	—	—
Level of fidelity enactment—performance	**Pre** ^d^	**Mid** ^d^	**Post** ^d^		***p***
FNs self‐rating^b^ sum score (inverse)		*n* = 18	*n* = 18		
Median [95% CI]^f^, comparison (*n* = 17)^g^	—	266 [254–282]	273.5 [263–281]	3 [−1–9]	0.265^i^
Min, Max	—	234, 291	225, 296	—	—
Low fidelity (range 60–180), *n* (%)	—	0 (0)	0 (0)	—	—
Moderate fidelity (range 181–252), *n* (%)	—	3 (16.7)	3 (16.7)	—	—
High fidelity (range > 252), *n* (%)	—	15 (83.3)	15 (83.3)	—	—
Component 1: Median [95% CI]^f^, comparison (*n* = 17)^g^	—	72 [68–75]	72 [67–74]	−1 [−3–1]	0.310^i^
Min, Max	—	62, 79	62, 77	—	—
Low fidelity (range 16–48), *n* (%)	—	0 (0)	0 (0)	—	—
Moderate fidelity (range 49–67), *n* (%)	—	4 (22.2)	5 (27.8)	—	—
High fidelity (range 68–80), *n* (%)	—	14 (77.8)	13 (72.2)	—	—
Component 2: Median [95% CI]^f^, comparison (*n* = 17)^g^	—	123 [117–132]	129 [124–131]	3 [−1–7]	0.026^i^
Min, Max	—	106, 136	102, 140	—	—
Low fidelity (range 28–84), *n* (%)	—	0 (0)	0 (0)	—	—
Moderate fidelity (range 85–118), *n* (%)	—	6 (33.3)	3 (16.7)	—	—
High fidelity (range 119–140) *n* (%)	—	12 (66.7)	15 (83.3)	—	—
Component 3: Median [95% CI]^f^, comparison (*n* = 17)^g^	—	73 [69–77]	75 [68–78]	1 [−1–3]	0.333^i^
Min, Max	—	62, 79	61, 80	—	—
Low fidelity (range 16–48), *n* (%)	—	0 (0)	0 (0)	—	—
Moderate fidelity (range 49–67), *n* (%)	—	4 (22.2)	3 (16.7)	—	—
High fidelity (range 68–80), *n* (%)	—	14 (77.8)	15 (83.3)	—	—
Observation of FNs^c^					
Component 1: *n* = 1, single observation	—	17 (17)	—	—	—
Low fidelity (range 33–48), *n* (%)	—	0 (0)	—	—	—
Moderate fidelity (range 23–32), *n* (%)	—	0 (0)	—	—	—
High fidelity (range 16–22) *n* (%)	—	1 (100)	—	—	—
Component 2: *n* = 4, Median (Min, Max)	—	29 (28, 33)	—	—	—
Low fidelity (range 58–84), *n* (%)	—	0 (0)	—	—	—
Moderate fidelity (range 40–57), *n* (%)	—	0 (0)	—	—	—
High fidelity (range 28–39) *n* (%)	—	4 (100)	—	—	—
Component 3: *n* = 5, Median (Min, Max)	—	16 (16, 17)	—	—	—
Low fidelity (range 33–48), *n* (%)	—	0 (0)	—	—	—
Moderate fidelity (range 23–32), *n* (%)	—	0 (0)	—	—	—
High fidelity (range 16–22) *n* (%)	—	5 (100.0)	—	—	—
FNs availability and capacity					
Statement 1. Availability, score range 1–5: *n* = 25, Median (Min, Max)	—	4 (3, 5)	—	—	—
Low fidelity (score 1 and 2), *n* (%)	—	0 (0)	—	—	—
Moderate fidelity (score 3 and 4), *n* (%)	—	15 (60.0)	—	—	—
High fidelity (score 5), *n* (%)	—	10 (40.0)	—	—	—
Statement 2. Capacity, score range 1–5: *n* = 15, Median (Min, Max)	—	5 (1, 5)	—	—	—
Low fidelity (score 1 and 2), *n* (%)	—	3 (20.0)	—	—	—
Moderate fidelity (score 3 and 4), *n* (%)	—	4 (26.7)	—	—	—
High fidelity (score 5), *n* (%)	—	8 (53.3)	—	—	—

Abbreviations: FN, Family Nurse; FNPS, Family Nurse Practice Skills; FSI, family support intervention; ICU, intensive care unit.

^a^ FNPS inverse scale range = 1.0–5.0 (lower scores indicate higher performance).

^b^ Self‐rating scale range 60–300, component 1: 16–80, component 2: 28–140, component 3: 16–80.

^c^ Observation inverse scale range per component: 1&3: 16–48, component 2: 28–84 (lower scores indicate higher performance).

^d^ Pre = before FSI implementation, Mid = 3–9 months after FSI start, Post = 18–24 months after FSI start.

^e^ Wilson's confidence interval (proportion).

^f^ Exact confidence interval of the median based on the binomial distribution.

^g^ Hodges–Lehmann location shift estimate of the paired median difference incl. 95% CI.

^h^ Friedman test of the overall null hypothesis between time points for complete cases.

^i^ Wilcoxon Signed‐Rank test of complete cases, including Holm‐Bonferroni correction for multiple pairwise comparisons of repeated measurement of an endpoint for at least three timepoints.

#### 5.2.2 Family Nurses' Skills

FNs' skills, as measured by the FNPS, were completed by 22 (100%), 17 (77%) and 15 (68%) FNs at timepoint *pre*, *mid* and *post*, respectively.

The overall FNPS sum score showed significant improvement over time, see Table 3. The overall null hypothesis between time points was rejected with *p* = 0.001, and pairwise comparison showed significant differences between timepoint *pre* and *mid* (*p* = 0.006; median difference −0.4) and between *pre* and *post* (*p* = 0.012; median difference −0.5), and non‐significant differences between *mid* and *post* (*p* = 0.304; median difference −0.1), indicating a steeper learning curve by FNs during the first 9 months of FSI implementation. Median scores per timepoint amounted to 2.0 (95% CI 1.7–2.4), 1.7 (95% CI 1.4–2.0) and 1.6 (95% CI 1.3–2.0) for timepoints *pre, mid and post*, respectively. Categorization into three fidelity levels showed low fidelity in two FNs (9.1%) with FNPS sum scores > 3.0 at timepoint *pre* and no low fidelity scores at timepoint *mid* and *post*. Moderate fidelity (FNPS scores between 1.9–3.0) was present in 13 FNs (59.1%) at timepoint *pre* and decreased to seven (41.2%) and five (33.3%) FNs at timepoint *mid* and *post*. High fidelity (FNPS scores ≤ 1.8) was present in seven FNs (31.8%) at timepoint *pre*, and in 10 (58.8%) and 10 (66.6%) FNs at timepoint *mid* and *post*, respectively.

### 5.3 Fidelity Enactment

#### 5.3.1 Family Nurses' FSI Performance—Self Rating

FNs' performance of the FSI, as assessed with the self‐rating tool by 18 FNs at timepoint *mid* and 18 FN at *post*‐implementation, was found to be rather high at both timepoints, with no significant increase over time with median scores of *mid* 266 (95% CI 254–282) and *post* 273.5 (95% CI 263–281), (*p* = 0.265; median difference 3.0), scale range 60–300, see Table 3. When analysing FSI components separately, component 2—*supporting*, including FN activities such as assessing family situation and resources and offering psychoeducation and relationship‐focused interventions through therapeutic conversations, significantly increased over time with median scores of *mid* 123 (95% CI 117–132) versus *post* 129 (95% CI 124–131), scale range 28–140, (*p* = 0.026; median difference 3.0). Component 1 (*engaging and liaising*) and 3 (*communicating*) did not show significant differences between timepoint *mid* and *post* with medians of component 1: 72 (95% CI 68–75) and 72 (95% CI 67–74), (*p* = 0.310; median difference −1), and component 3: 73 (95% CI 69–77) and 75 (95% CI 68–78), (*p* = 0.333; median difference 1.0), scale range 16–80. Fidelity levels were high with 15 FNs (83.3%) scoring 80%–100% of the scale range and three FNs (16.7%) scoring moderate fidelity with scores between 51% and 79% at timepoint *mid* and *post*, respectively, see Table 3.

#### 5.3.2 Family Nurses' FSI Performance—Observation

In total, eight structured observations, one at each participating ICU were performed at timepoint *mid*. FSI component 1—*liaising and engaging* was observed once, FSI component 2—*supporting* was observed in four instances and FSI component 3—*communication* in five. All observed FSI components scored high on performance with a median inverse sum score of 17 (score range 17), scale range 16–48 for component 1, a median of 29 (score range 28–33), scale range 28–84 for component 2 and a median of 16 (score range 16–17), scale range 16–48 for component 3, see Table 3. Indicating that fidelity scores were all high.

#### 5.3.3 Family Nurses' Availability and Capacity for Intervention Delivery

Statement 1 on the availability of FNs during 5 days per week was completed by 25 participants, including 15 FNs and 10 local ICU study team members (managers, implementation support and study coordinators). Their median score was 4 (score range 3–5), see Table 3. In total, 15 participants (60%) scored 3 or 4, indicating moderate fidelity and 10 participants (40%) scored 5, indicating high fidelity on FNs availability. Statement 2 on FNs' capacity to provide the FSI was completed by 15 FNs with a median score of 5 (score range 1–5). Three FNs (20%) rated their capacity with a score of 1 or 2 (low fidelity), four (26.7%) rated it with a score of 3 or 4 (moderate fidelity) and eight (53.3%) rated it with a score of 5 (high fidelity).

### 5.4 Family Nurses' Experiences With the FSI

Three main themes and subthemes related to FN experiences with the FSI emerged from the data: (1) role development, (2) role enactment and (3) role support.

#### 5.4.1 Theme 1. Role Development

To develop their role, FNs noticed the FSI training in which theory and practice were combined as well as previous life and work experience, reflection and exchange with other FNs, and building resilience through practical experiences as important contributors.

##### 5.4.1.1 Skill Development

Skill development refers to how the FSI training, together with prior professional experience and learning by doing, contributed to the development of FNs' communication skills and confidence in their role.

The 5‐day FSI‐training supported skill development and empowerment of FNs. Training helped FNs to learn how to interact with families, as one FN described: ‘Well, the teacher always helped me a lot. That was… for me, it was really in the back of my mind, everything… that you just can't solve problems and that sometimes families just talk or (.) and then you can just let them talk…’ (*FG 2*). In addition, individual life and work experience formed a crucial foundation for role development, as explained by a nurse: ‘I'm not just talking about the training we had ‐ because it was good, but I think… especially at the beginning, (.) Rather, it was the professional experience I already had, the whole package and a little bit of experience, what it means to be a patient in ICU…, I feel that's probably more valuable than just the training…’ (*FG 1*). Initially, FNs often felt unsure but became more confident through practical experience, by ‘learning by doing’: ‘…because it was all so theoretical what you learned from the training. And then you didn't really know how to put it into practice … I thought, how am I supposed to put that into practice… And you just had to do that a few times to feel more confident’. (*FG 2*).

##### 5.4.1.2 Reflection and Exchange

Reflection and exchange highlights how recognition of personal and role limitations, combined with structured opportunities for discussion, contributed to FNs' role development. This subtheme emphasizes the importance of sharing experiences and discussing cases, while also noting that the format of these exchanges can influence their effectiveness.

Recognition of and experiencing personal and role limitations was mentioned as another important contributor to the FN role development: ‘And because… we have certain things that we can't do, and it's also important to recognize that, because otherwise you put even more pressure on yourself… And just saying: look, this is as far as I can go… That took a huge weight off my shoulders’. (*FG 2*). Reflection and exchange between FNs fostered role‐finding, provided an opportunity for them to discuss approaches to handle family matters in‐depth and keep each other informed about ongoing activities. One FN said: ‘The two of us met up with our implementation support person, mostly on an ad hoc basis, and had a kind of case conference to discuss cases’. (*FG 1*) and another nurse said: ‘We got off to a good start and exchanged a lot of information. And then we also… we wrote reports sometimes, and we also sent each other emails with a summary of the most important things that had happened’. (*FG 1*). Regular case conferences offered by the research team were seen as a valuable contribution to exchange and reflection on specific family situations, as mentioned by one FN: ‘Case discussions were also incredibly helpful for me. I have to say that just hearing about the experiences surrounding them and simply having the exchange is really nice, yes’ (*FG 1*). However, not all appreciated the online format and found it as constraining for the discussion and exchange: ‘I find case discussions, reflection, and so online, but that's just not my thing. It's simply the closeness that I actually need that creates trust and connection. That doesn't happen for me…’ (*FG 2*).

##### 5.4.1.3 Building Resilience

Building resilience captures the challenges FNs faced in managing complex family situations while maintaining professional boundaries. This subtheme highlights how FNs developed coping strategies, set limits, and relied on team support to balance the emotional demands of their role with personal well‐being.

Building resilience in working with families and coping with complex family situations was highly challenging for many FNs. Family situations and needs were sometimes so intense that FNs tended to get drawn into them in a way that made it difficult to handle them, as one FN described: ‘Um… (.) in conversation, where it goes so deep, you often don't want to go that deep. You get really sucked in. (.) You have to be able to deal with that’. (*FG1*). Others were able to see and set boundaries: ‘And I didn't really have any problems with setting boundaries. And when there were actual cases, I was able to talk about it with the team…’ (*FG 2*) or found alternatives to cope ‘… two or three family situations became so difficult, … then the other (FN) took over. And that worked out quite well… because then there's a whole different dynamic…’ (*FG 1*).

Many FN mentioned the challenge to regulate work hours with both FN and bedside tasks as illustrated by one FN: ‘It's easy to end up working a 12‐hour shift… And you might do that two or three times, and then you just reach your personal limit, I would say, of balancing yourself and living and maintaining a private life somewhere’. (*FG 1*).

#### 5.4.2 Theme 2. Role Enactment

Nursing team support, acceptance and collaboration within the interprofessional teams were factors influencing enactment of the FN role within established ICU structures.

##### 5.4.2.1 Team Hesitance and Acceptance

Team hesitance and acceptance refer to how FNs' integration of the novel FSI was shaped by team attitudes, including support, apprehension and competition within established ICU structures.

Delivering the novel FSI within established ICU structures posed challenges for FNs. Beside open attitudes and supportive team members, some FNs were confronted with feelings of apprehension within nursing teams, as one FN said: ‘…it's been very tightly controlled by certain colleagues: does she do her work at the bed first or is she going to do the study intervention now…’ (*FG 1*). Some FNs experienced a sense of competition within the team because of their new role: ‘On the one hand, it was expected: you look at the families, and on the other hand, you sense: ah, she's taking something away from us…’ (*FG 1*).

##### 5.4.2.2 Collaboration

Collaboration refers to how FNs navigated initial scepticism and gradually developed supportive interprofessional relationships, which were essential for delivering continuous family‐centred care.

Enacting the FN role in collaboration with the intensivists required initial work and effort. Some FNs experienced scepticism from interprofessional team members they collaborated with, which needed time and beneficial experiences with the FSI to turn into a supportive collaboration, ‘Where there was skepticism at the beginning: (.) yes, what do they want now, and yes, the medical studies are the ones that are good and count, and what they then actually noticed themselves (.) that it is a benefit…’ (*FG 1*). The support from the medical team was important for FNs to ensure continuity of family care. Enacting the FN role within the interprofessional team felt for some FNs like a balancing act to do good on both sides, as illustrated by one FN: ‘And (.) it's … I always felt it was a bit like walking a tightrope or performing a balancing act’. (*FG 2*).

#### 5.4.3 Theme 3. Role Support

##### 5.4.3.1 Team Internal Support

Team internal support highlights the crucial role of nurse and physician leadership in enabling FNs to develop and enact their role within the interprofessional ICU team. This subtheme emphasizes how leadership support – both explicit and implicit – helped define responsibilities, manage workload, and provide emotional and practical support, while the absence of such structures made role enactment more challenging.

Nurse and physician leadership support and commitment formed an important precondition for FNs to develop and enact their role within the interprofessional ICU team. In some situations, support went without saying, as mentioned by one FN: ‘We never formalized anything: like we must do it this way and now we must do it that way. It just happened that way… Instead, we had complete freedom…’ (*FG 2*). However, in other situations, FNs wished for more explicit support to enact their FN role: ‘And I firmly believe that when the management sets an example…, then the team also feels obliged to participate. So, I believe that this is practiced top down…’ (*FG 1*). Another FN said that the support of the physician chief of staff was an important precondition: ‘Um, at our place, I had the feeling that in finding the right role… was incredibly helpful for us, that from the medical side, our chief physician… gave us a position’ (*FG 1*). Leadership support was also welcome in framing the role, responsibilities and the workload for the FNs themselves, but also towards the team, as one FN said: ‘So I would prefer to have more defined pre‐conditions. Somehow, I think there would be more certainty… now I think we know the scope, we know the effort involved, the extent of what it does to you, also on an emotional level. (.) I think I would like to know that I have my space, I have the time, and that structures are in place’. (*FG 2*).

FNs expressed the burdensome and emotionally challenging situations they were confronted with and the importance of coaching and support within their teams to prevent them from taking the burden ‘home’. This was not always well established, and some FNs found it difficult dealing with it, as illustrated: ‘So I felt very uncertain. (.) Simply because we didn't have anyone to talk to, or rather, I didn't have anyone to talk to.’ (*FG 2*). Another FN emphasized on the responsibility of team leaders to organize this and said: ‘…We didn't have that supervision here. And I say, I don't want to know how many people here were affected by what they took home with them. I often brought this up, but it wasn't taken seriously.’ (*FG 2*).

##### 5.4.3.2 Team External Support

Team external support refers to how guidance from the research team and positive feedback from family members enhanced FNs' confidence, motivation and engagement in delivering the intervention.

External research team support by phone and the quick response to situations in which FNs needed support was valued. However, more presence on site would have been appreciated, as mentioned by one FN: ‘That you're there on site ‐ then you might be more likely to sense how discussions are going, whether there might be a little more need after all… Just drop by and see how things are going’. (*FG 2*). An important motivator for FNs was the appreciation and the positive feedback from family members who confirmed the meaningfulness and importance of their work, as mentioned: ‘I experienced it that way too, or that the relatives' feedback was what gave me the most energy.’ (*FG 1*) and another said, ‘At first, he (family member) rejected everything, but once he was able to open up, he really needed about two hours of conversation every day. (.) It showed me that the intervention works and does good. And it reaffirmed me in my role’. (*FG 2*).

### 5.5 Data Integration

In Table 4, main findings of the quantitative and qualitative data, and development of the meta‐inferences are displayed. Further interpretation of the findings is described in Section 6.

**TABLE 4:** Joint display table of qualitative and quantitative data with meta‐inferences.

Quantitative findings	Qualitative findings	Meta‐inferences
FNs' overall case conference attendance: 63.5% FNs level of fidelity at post: Low: 7 (31.8%) Moderate: 11 (50%) High: 4 (18.2%)	Theme 1. Role development—skill development: FSI training, together with prior professional experience and learning by doing, contributed to the development of FNs' communication skills and confidence in their role.	Fidelity receipt—family nurses' skills and learning trajectories: Not all FNs attended case conferences for reflection and exchange. FNs mentioned that not all FNs appreciated the online format, which may have affected the fidelity receipt. A steeper learning curve based on the FNPS was observed in the first 9 months after implementation, indicating a steeper learning curve in the early phase of FSI delivery and further skill development through practical experience. Through experiences and reflection, confidence and emotional resilience were built.
FNPS: Moderate to high at pre, mid and post and increased over time (*p* = < 0.001). FNs levels of fidelity pre, mid and post: Low: 2 (9.1%) at pre and 0 at mid and post. Moderate: 13 (59.1%) at pre, 7 (41.2%) mid, 5 (33.3%) post. High: 7 (31.8%) at pre, 10 (58.8%) at mid, 10 (66.6%) post.	Theme 1. Role development—Reflection and exchange: Recognition of personal and role limitations and structured opportunities for reflection support FNs' role development. Sharing experiences and discussing cases is important, however the format should be considered, for example, online or in‐person.	
	Theme 1. Role development—Building resilience: FNs experience challenges in managing complex family situations and setting professional boundaries. The FN role comes with emotional demands affecting personal well‐being. Coping strategies, team support and setting limits, supported FNs to cope with demanding situation.	
FNs FSI self‐rating: High at mid and post, no significant increase over time (*p* = 0.265). FSI component (2), increased between mid and post (*p* = 0.26). Components 1 and 3 did not show differences. FNs level of fidelity: Low: 0 Moderate: 3 (16.7%) High: 15 (83.3%)	Theme 2. Role enactment—Team hesitance and acceptance: Nursing team support, acceptance and collaboration within the interprofessional teams influenced the FN role enactment.	Fidelity enactment—family nurses' FSI performance and opportunity for delivery: FNs performance based on the self‐rating was high across all three intervention components. Supporting families (component 2) increased significantly over time. This component requires a high level of relational skills and family systems nursing competencies which is also reflected in the increased FNPS skills. The observations high FSI performance and supported the self‐rating findings. FSI enactment was influenced by the ICU context, for example, medical and nursing team acceptance of the FN role. Leadership support is crucial in developing and enacting the FN role, as well as positive feedback from families. The availability and capacity of FN was considered good and an important precondition for FSI enactment.
FN FSI Observations: High for all FSI components, no differentiation between components. FNs Level of fidelity: high for all observed components.	Theme 2. Role enactment – Collaboration: FNs experienced initial scepticism, but collaborations developed to supportive interprofessional collaborations to support families.	
FNs level of fidelity—Availability: Low: 0 Moderate: 15 (60%) High: 10 (40%) FNs level of fidelity—Capacity: Low: 3 (20%) Moderate: 4 (26.7%) High: 8 (53.3%)	Theme 3. Role support—Team internal support: Nurse‐ and physician leadership is important to facilitate FNs in their role enactment with the interprofessional ICU context. Leadership support should focus on managing workload, providing practical and emotional support and structure responsibilities.	
	Theme 3. Role support—Team external support: External support from the research team and positive feedback from family members support FNs in delivering the FSI.	

Abbreviations: FN, family nurse; FNPS, Family Nurse Practice Scale; FSI, family support intervention; ICU, intensive care unit.

## 6 Discussion

This mixed‐methods evaluation assessed fidelity *receipt* and *enactment* and examined critical care FNs' experiences with delivering a novel FSI within the context of the FICUS trial (Naef et al. 2025; Riguzzi et al. 2026). Overall, findings indicate that FNs achieved moderate to high fidelity *receipt* and high fidelity *enactment* of the FSI, while qualitative data provide important insight into the mechanisms through which skills were developed and enacted within the established ICU structures.

### 6.1 Fidelity Receipt: Family Nurses' Skills and Learning Trajectories

Fidelity *receipt*, operationalized through case conference attendance and self‐reported family nursing skills, was overall moderate to high. Skills improved significantly from pre to mid and from pre to post, but no significant improvement from mid to post could be proven. This pattern suggests a relatively steep initial learning curve during the first 9 months of initial implementation, followed by consolidation and maintenance of skills. Such learning trajectories are consistent with prior implementation studies of complex nursing interventions, where early training and early practice opportunities yield the greatest increase in competence, while later phases support refinement and contextual application of skills (Ginsburg et al. 2020; Guerbaai et al. 2023).

Qualitative findings help explain these quantitative patterns. FNs described the FSI training as a critical foundation for understanding family systems nursing principles but emphasized that true skill acquisition occurred through experiential learning, reflection and peer exchange. This finding aligns with nursing education research showing that simulation, structured clinical experiences and reflective activities enhance clinicians' readiness to apply knowledge across varied situations and support deeper integration of competencies into practice (López‐Medina et al. 2025; Miraglia and Asselin 2015). From this perspective, fidelity *receipt* should be viewed not merely as knowledge acquisition but as the internalization of FSI principles and the development of clinical experience required to apply these principles tailored to various family situations.

The finding that some FNs attended fewer than half of the case conferences, and that online formats were not appreciated by all FNs, suggests that while structured learning opportunities were considered important for reflection and exchange, their format and perceived relevance influence the level of engagement and may affect fidelity *receipt*. Studies indicate that reflective and case‐based learning particularly benefit from face‐to‐face or blended formats, as these allow for greater interaction and social presence (Chim et al. 2024; Regmi and Jones 2020).

These findings suggest that, in the context of the FSI, fidelity *receipt* emerges from a blended process of training, practice and reflection in peer exchange. Structured educational input provides a basis for early competence development, and ongoing practice experiences consolidate and deepen professional skills. In this perspective, fidelity *receipt* should be considered as a reflective developmental process in which nurses progressively adopt FSI principles and adapt them to family situations.

### 6.2 Fidelity Enactment: Family Nurses' FSI Performance and Opportunity for Delivery

Fidelity *enactment* of the FSI was consistently high, as demonstrated by self‐developed, non‐validated and therefore exploratory self‐assessments and observational data. FNs reported high levels of performance across all three intervention components, with a significant increase over time observed only for FSI component 2 – *supporting*. This component, which includes therapeutic conversations, psychoeducation and relationship‐focused interventions, requires the highest level of relational skill and family system nursing competencies. Its improvement over time likely reflects growing confidence with challenging family encounters and increased proficiency in applying family systems approaches in practice. Previous family nursing research similarly suggests that relational and therapeutic competencies develop progressively through repeated clinical encounters and reflection (Östlund et al. 2015; Shajani and Snell 2023). The absence of significant changes in component 1 – *engaging & liaising* and 3 – *communicating* may indicate that these activities were already well integrated into FNs' competencies, or that the self‐assessment instrument lacked sensitivity to detect refinements of the components, or that the sample was too small and power therefore insufficient to detect small changes. The pattern of findings suggests that enactment of the FSI was already well established, with further improvement occurring specifically in the supporting component 2. This is in line with research showing that enactment measures capture improvements more clearly in complex, decision‐based activities than in routine or standard tasks (Wiltsey Stirman et al. 2019). Despite the limited number of observations and their uneven distribution across the FSI components, observational data corroborated the high degree of accuracy with which the FSI was enacted, supporting the validity of the self‐reported enactment findings.

Importantly, high fidelity *enactment* was observed despite variability in reported FN availability and capacity for FSI delivery. While most participants reported moderate to high availability of FNs, a substantial proportion indicated limited capacity to deliver the FSI consistently. This finding aligns with literature indicating that high‐quality enactment of complex nursing interventions can be sustained at the individual level even in the presence of structural constraints, particularly when professionals have developed strong intervention ownership and clinical experience (May et al. 2018; Proctor et al. 2011). At the same time, persistent limitations in delivery capacity may threaten long‐term sustainability, underscoring the importance of organizational support to maintain opportunities for enactment over time (Azoulay et al. 2024).

### 6.3 Role Development and Enactment as Mechanisms of Fidelity

The qualitative themes of role development, role enactment and role support highlight key mechanisms underlying fidelity receipt and enactment. Role development was shaped by a combination of training, prior professional and life experience, reflective practice and peer support. FNs' experiences highlight that developing competence in family systems care requires not only technical skills but also emotional resilience, boundary‐setting and self‐awareness.

Role enactment was strongly influenced by the social and organizational context of the ICUs. While some nursing and medical teams immediately accepted the FN role, others exhibited hesitance or perceived competition. These dynamics impacted the extent to which FNs could fulfil their role, demonstrating that fidelity enactment is dependent on interprofessional relationships and such within the nursing team rather than being an individual FN attribute alone. Over time, positive experiences with the FSI appeared to mitigate scepticism among individual staff members by experiencing the FSI value in clinical practice. In addition, leadership support emerged as a central enabling factor for both fidelity receipt and enactment. Explicit endorsement by nursing and medical leaders helped legitimize and position the FN role, allowed protected time for FSI activities, and clarified expectations within teams. Conversely, ambiguous role definitions and insufficient emotional support structures left some FNs feeling uncertain or overburdened. These findings align with the literature emphasizing leadership engagement and supportive infrastructure as critical determinants of implementation and integration success for complex interventions such as the FSI (Skivington, Matthews, et al. 2024; Walton 2024). Furthermore, the continuous learning collaborative for FNs was highly valued; however, participants expressed a desire for more on‐site involvement from the research team and additional internal coaching. This indicates that while centralized support is beneficial, locally embedded mentorship and supervision may be especially important for sustaining the emotionally demanding role of FNs in critical care. Research on coaching in nursing practice indicates that structured coaching and mentorship programmes enhance clinical performance, role effectiveness, confidence and support for staff, suggesting that workplace‐embedded coaching could strengthen both professional competence and emotional resilience (Richardson et al. 2023).

### 6.4 Strength and Limitations of the Work

This study contributes novel evidence on the implementation of nurse‐led FSIs by being among the first to empirically link fidelity *receipt* and *enactment* to FN role development and performance in a critical care context. A key strength is its longitudinal, convergent mixed‐methods design, which allowed for an in‐depth examination of fidelity processes over time and the integration of quantitative and qualitative perspectives. This study also has limitations. The relatively small sample size and limited number of FSI observations restrict generalizability and the precision of fidelity *receipt* and *enactment* assessment. Non‐significant differences are not proof of absence of differences in the population. Although the self‐reported measure may be subject to social desirability bias, triangulation with observational and qualitative data mitigates this concern. However, we were unable to perform a confirmatory analysis due to different nurse‐family interactions observed and self‐rated. In addition, the developed instruments for the self‐rating of FSI performance and observation of FSI delivery were not validated and therefore exploratory, and we may have overlooked mechanisms contributing to fidelity *enactment*.

### 6.5 Recommendations for Practice and Further Research

Our findings have implications for both clinical practice and future research. For practice, organizations implementing nurse‐led FSI should prioritize structured training, protected time, clear role definitions and locally embedded mentorship or coaching to support professional competence and emotional resilience. Leadership engagement and supportive infrastructure are critical to enable nurses to enact complex FSIs consistently. For research, further studies should explore how fidelity *receipt* and *enactment* relate to family‐ and patient‐level outcomes and refine methods for assessing fidelity in complex nursing interventions. In this context, the recent development and validation of the Overall Fidelity Enactment Scale for Complex Interventions (OFES‐CI) by Ginsburg et al. (2024) represents a promising step forward by providing a practical tool to rate fidelity enactment using structured observations and expert assessments, with good reliability demonstrated in pilot testing among nursing home teams. Future work could adapt and test this instrument in the context of FSIs.

## 7 Conclusion

FNs who acted as intervention nurses in the context of the FICUS trial demonstrated moderate to high fidelity *receipt* of the FSI and developed their skills through structured training, experiential learning and reflection. Fidelity *enactment* was consistently high, with FNs successfully delivering core intervention components and activities, particularly relational and supportive practices. FNs' experiences of the FSI were shaped not only by individual competency, but also by interprofessional relationships, leadership support and organizational context. FNs emphasized that developing competence required practical, emotional and interprofessional skills, emotional resilience and boundary‐setting, while *enactment* depended on team acceptance, clear role definitions and protected time. These findings indicate that nurse‐led FSIs can be integrated into ICU practice when considering the importance of ongoing mentorship, protected time and supportive structures to facilitate both skill development and FSI *enactment*.

## Author Contributions

**Lotte Verweij:** conceptualization, investigation, writing – original draft, methodology, validation, visualization, software, formal analysis. **Saskia Oesch:** investigation, methodology, validation, writing – review and editing, formal analysis, conceptualization. **Marco Riguzzi:** conceptualization, investigation, methodology, validation, writing – review and editing, formal analysis. **Simone Sutter:** validation, writing – review and editing, project administration, investigation. **Rahel Naef:** conceptualization, investigation, funding acquisition, methodology, validation, writing – review and editing, formal analysis, supervision, resources.

## Funding

The study is funded by the Swiss National Science Fund (SNSF, No. 33IC30_198778/1).

## Disclosure

Statistician: There is a statistician (M.R.) on the author team.

## Ethics Statement

The FICUS trial was approved by the responsible Swiss cantonal ethics committee (No. 2021‐02300). For this embedded mixed‐methods study, the lead ethics committee waived the need for additional ethical approval (BASEC‐Nr. Req‐2023‐01026). Participating family nurses provided informed consent for study participation.

## Conflicts of Interest

The authors declare no conflicts of interest.

## Supporting information


**Supplementary File S1:** Good reporting of a mixed‐methods study (GRAMMS).

**Supplementary File S2:** Self‐developed family support intervention self‐rating and observation tool(1).

**Supplementary File S3:** Interview guide (condensed version).

## Data Availability

The data that support the findings of this study are available on request from the corresponding author. The data are not publicly available due to privacy or ethical restrictions.

## References

[nop270908-bib-0001] Albers, B. , L. Verweij , K. Blum , et al. 2024. “Firm, Yet Flexible: A Fidelity Debate Paper With Two Case Examples.” Implementation Science 19, no. 1: 79. 10.1186/s13012-024-01406-3. PMC11619306 39639379

[nop270908-bib-0002] Azoulay, É. , N. Kentish‐Barnes , C. Boulanger , et al. 2024. “Family Centeredness of Care: A Cross‐Sectional Study in Intensive Care Units Part of the European Society of Intensive Care Medicine.” Annals of Intensive Care 14, no. 1: 77. 10.1186/s13613-024-01307-0. PMC11109056 38771395

[nop270908-bib-0003] Azoulay, É. , F. Pochard , J. Nelson , M. Herridge , S. Jaber , and N. Kentish‐Barnes . 2025. “Healthcare‐Associated Family Mental Injury (HILLY).” American Journal of Respiratory and Critical Care Medicine 212: 718–720. 10.1164/rccm.202501-0313VP. 41072432

[nop270908-bib-0004] Bell, J. M. , and L. M. Wright . 2021. Illness Beliefs: The Heart of Healing in Families and Individuals. Revised 3rd ed. 4th Floor Press.

[nop270908-bib-0005] Bellg Borrelli, B. , B. Resnick , J. Hecht , et al. 2004. “Enhancing Treatment Fidelity in Health Behavior Change Studies: Best Practices and Recommendations From the NIH Behavior Change Consortium.” Health Psychology 23, no. 5: 443–451. 10.1037/0278-6133.23.5.443. 15367063

[nop270908-bib-0006] Bohart, S. , A. H. Nielsen , J. L. Sørensen , et al. 2024. “Establishing Consensus on Patient‐ and Family‐Centered Care in Adult Intensive Care Units: A Delphi Survey.” Journal of Critical Care 84: 154859. 10.1016/j.jcrc.2024.154859. 39003924

[nop270908-bib-0007] Borrelli, B. , D. Sepinwall , D. Ernst , et al. 2005. “A New Tool to Assess Treatment Fidelity and Evaluation of Treatment Fidelity Across 10 Years of Health Behavior Research.” Journal of Consulting and Clinical Psychology 73: 852–860. 10.1037/0022-006X.73.5.852. 16287385

[nop270908-bib-0008] Carroll, C. , M. Patterson , S. Wood , A. Booth , J. Rick , and S. Balain . 2007. “A Conceptual Framework for Implementation Fidelity.” Implementation Science 2, no. 1: 40. 10.1186/1748-5908-2-40. PMC2213686 18053122

[nop270908-bib-0009] Carter, N. , D. Bryant‐Lukosius , A. DiCenso , J. Blythe , and A. J. Neville . 2014. “The Use of Triangulation in Qualitative Research.” Oncology Nursing Forum 41, no. 5: 545–547. 10.1188/14.Onf.545-547. 25158659

[nop270908-bib-0010] Chim, H. Q. , D. H. J. M. Dolmans , M. G. A. Oude Egbrink , and H. H. C. M. Savelberg . 2024. “Experiences of Face‐To‐Face and Online Collaborative Learning Tutorials: A Qualitative Community of Inquiry Approach.” Education and Information Technologies 29, no. 14: 18561–18589. 10.1007/s10639-024-12533-1.

[nop270908-bib-0011] Coventry, A. , M. Gerdtz , E. McInnes , J. Dickson , and P. Hudson . 2023. “Supporting Families of Patients Who Die in Adult Intensive Care: A Scoping Review of Interventions.” Intensive & Critical Care Nursing 78: 103454. 10.1016/j.iccn.2023.103454. 37253283

[nop270908-bib-0012] Creswell, J. W. 2021. A Concise Introduction to Mixed Methods Research. 2nd ed. SAGE Publications Inc.

[nop270908-bib-0013] Creswell, J. W. , and V. L. Plano Clark . 2018. Designing and Conducting Mixed Methods Research. 3rd ed. SAGE Publications Inc.

[nop270908-bib-0014] Danford, C. A. , K. Mooney‐Doyle , J. A. Deatrick , et al. 2024. “Building Family Interventions for Scalability and Impact.” Journal of Family Nursing 30, no. 2: 94–113. 10.1177/10748407241231342. 38629802

[nop270908-bib-0015] Duong, J. , G. Wang , G. Lean , D. Slobod , and M. Goldfarb . 2024. “Family‐Centered Interventions and Patient Outcomes in the Adult Intensive Care Unit: A Systematic Review of Randomized Controlled Trials.” Journal of Critical Care 83: 154829. 10.1016/j.jcrc.2024.154829. 38759579

[nop270908-bib-0016] Dusenbury, L. , R. Brannigan , M. Falco , and W. B. Hansen . 2003. “A Review of Research on Fidelity of Implementation: Implications for Drug Abuse Prevention in School Settings.” Health Education Research 18, no. 2: 237–256. 10.1093/her/18.2.237. 12729182

[nop270908-bib-0017] Elo, S. , and H. Kyngäs . 2008. “The Qualitative Content Analysis Process.” Journal of Advanced Nursing 62, no. 1: 107–115. 10.1111/j.1365-2648.2007.04569.x. 18352969

[nop270908-bib-0018] Erlingsson, C. , and P. Brysiewicz . 2017. “A Hands‐On Guide to Doing Content Analysis.” African Journal of Emergency Medicine 7, no. 3: 93–99. 10.1016/j.afjem.2017.08.001. PMC6234169 30456117

[nop270908-bib-0019] Fetters, M. D. , L. A. Curry , and J. W. Creswell . 2013. “Achieving Integration in Mixed Methods Designs‐Principles and Practices.” Health Services Research 48, no. 6 Pt 2: 2134–2156. 10.1111/1475-6773.12117. PMC4097839 24279835

[nop270908-bib-0020] Fixsen, D. L. 2025. “Fidelity, Not Adaptation, Is Essential for Implementation.” Frontiers in Health Services 5: 1575179. 10.3389/frhs.2025.1575179. PMC12061988 40353253

[nop270908-bib-0021] Garbacz, L. L. , D. M. Brown , G. A. Spee , A. J. Polo , and K. S. Budd . 2014. “Establishing Treatment Fidelity in Evidence‐Based Parent Training Programs for Externalizing Disorders in Children and Adolescents.” Clinical Child and Family Psychology Review 17, no. 3: 230–247. 10.1007/s10567-014-0166-2. 24706293

[nop270908-bib-0022] Gearing, R. E. , N. El‐Bassel , A. Ghesquiere , S. Baldwin , J. Gillies , and E. Ngeow . 2011. “Major Ingredients of Fidelity: A Review and Scientific Guide to Improving Quality of Intervention Research Implementation.” Clinical Psychology Review 31, no. 1: 79–88. 10.1016/j.cpr.2010.09.007. 21130938

[nop270908-bib-0023] Ginsburg, L. , M. Hoben , W. Berta , et al. 2024. “Development and Validation of the Overall Fidelity Enactment Scale for Complex Interventions (OFES‐CI).” BMJ Quality and Safety 33, no. 2: 98–108. 10.1136/bmjqs-2023-016001. PMC10850642 37648435

[nop270908-bib-0024] Ginsburg, L. R. , M. Hoben , A. Easterbrook , et al. 2020. “Examining Fidelity in the INFORM Trial: A Complex Team‐Based Behavioral Intervention.” Implementation Science 15, no. 1: 78. 10.1186/s13012-020-01039-2. PMC7493316 32938481

[nop270908-bib-0025] Ginsburg, L. R. , M. Hoben , A. Easterbrook , R. A. Anderson , C. A. Estabrooks , and P. G. Norton . 2021. “Fidelity Is Not Easy! Challenges and Guidelines for Assessing Fidelity in Complex Interventions.” Trials 22, no. 1: 372. 10.1186/s13063-021-05322-5. PMC8164256 34051830

[nop270908-bib-0026] Graneheim, U. H. , B. M. Lindgren , and B. Lundman . 2017. “Methodological Challenges in Qualitative Content Analysis: A Discussion Paper.” Nurse Education Today 56: 29–34. 10.1016/j.nedt.2017.06.002. 28651100

[nop270908-bib-0027] Guerbaai, R. A. , S. DeGeest , L. L. Popejoy , et al. 2023. “Evaluating the Implementation Fidelity to a Successful Nurse‐Led Model (INTERCARE) Which Reduced Nursing Home Unplanned Hospitalisations.” BMC Health Services Research 23, no. 1: 138. 10.1186/s12913-023-09146-8. PMC9910256 36759902

[nop270908-bib-0028] Hwang, D. Y. , S. J. W. Oczkowski , K. Lewis , et al. 2025. “Society of Critical Care Medicine Guidelines on Family‐Centered Care for Adult ICUs: 2024.” Critical Care Medicine 53, no. 2: e465–e482. 10.1097/ccm.0000000000006549. PMC13537439 39982184

[nop270908-bib-0029] Kiwanuka, F. , N. Sak‐Dankosky , Y. H. Alemayehu , R. C. Nanyonga , and T. Kvist . 2022. “The Evidence Base of Nurse‐Led Family Interventions for Improving Family Outcomes in Adult Critical Care Settings: A Mixed Method Systematic Review.” International Journal of Nursing Studies 125: 104100. 10.1016/j.ijnurstu.2021.104100. PMC8560087 34736074

[nop270908-bib-0030] Lebel, V. , and S. Charette . 2021. “Nursing Interventions to Reduce Stress in Families of Critical Care Patients: An Integrative Review.” Critical Care Nurse 41, no. 1: 32–44. 10.4037/ccn2021188. 33560437

[nop270908-bib-0031] López‐Medina, I. M. , S. Sanz‐Martos , C. Álvarez‐García , C. Hueso‐Montoro , A. A. Esteban‐Burgos , and C. Álvarez‐Nieto . 2025. “Acquisition of Nursing Competencies Using Advanced Clinical Simulation and Virtual Reality: A Pre‐ and Post‐Intervention Study.” Teaching and Learning in Nursing 21: e961–e967. 10.1016/j.teln.2025.09.014.

[nop270908-bib-0032] MacEachern, L. , L. R. Ginsburg , M. Hoben , et al. 2023. “Developing a Tool to Measure Enactment of Complex Quality Improvement Interventions in Healthcare.” BMJ Open Quality 12, no. 1: e002027. 10.1136/bmjoq-2022-002027. PMC9923287 36754540

[nop270908-bib-0033] MAXQDA . 2024. https://www.nvivo.de/.

[nop270908-bib-0034] May, C. R. , A. Cummings , M. Girling , et al. 2018. “Using Normalization Process Theory in Feasibility Studies and Process Evaluations of Complex Healthcare Interventions: A Systematic Review.” Implementation Science 13, no. 1: 80. 10.1186/s13012-018-0758-1. PMC5992634 29879986

[nop270908-bib-0035] Miraglia, R. , and M. E. Asselin . 2015. “Reflection as an Educational Strategy in Nursing Professional Development: An Integrative Review.” Journal for Nurses in Professional Development 31, no. 2: 62–72; quiz E66. 10.1097/nnd.0000000000000151. 25790356

[nop270908-bib-0036] Naef, R. , J. Ernst , M. Müeller , and G. Schmid‐Mohler . 2021. “Translation and Psychometric Validation of the German Version of the Family Nursing Practice Scale (FNPS).” Journal of Family Nursing 27, no. 1: 34–42. 10.1177/1074840720969387. 33183149

[nop270908-bib-0037] Naef, R. , M. Filipovic , M.‐M. Jeitziner , et al. 2022. “A Multicomponent Family Support Intervention in Intensive Care Units: Study Protocol for a Multicenter Cluster‐Randomized Trial (FICUS Trial).” Trials 23, no. 1: 533. 10.1186/s13063-022-06454-y. PMC9235279 35761343

[nop270908-bib-0038] Naef, R. , M. M. Jeitziner , M. Riguzzi , et al. 2025. “Nurse‐Led Family Support Intervention for Families of Critically Ill Patients: The FICUS Cluster Randomized Clinical Trial.” JAMA Internal Medicine 185, no. 9: 1138–1149. 10.1001/jamainternmed.2025.3406. PMC12558129 40720110

[nop270908-bib-0039] O'Cathain, A. , E. Murphy , and J. Nicholl . 2008. “The Quality of Mixed Methods Studies in Health Services Research.” Journal of Health Services Research & Policy 13, no. 2: 92–98. 10.1258/jhsrp.2007.007074. 18416914

[nop270908-bib-0040] Oesch, S. , L. Verweij , L. Clack , T. Finch , M. Riguzzi , and R. Naef . 2023. “Implementation of a Multicomponent Family Support Intervention in Adult Intensive Care Units: Study Protocol for an Embedded Mixed‐Methods Multiple Case Study (FICUS Implementation Study).” BMJ Open 13, no. 8: e074142. 10.1136/bmjopen-2023-074142. PMC10414125 37553195

[nop270908-bib-0041] Oesch, S. , L. Verweij , M. Riguzzi , T. Finch , L. Clack , and R. Naef . 2025. “Integrating a Family Support Intervention in Intensive Care Units: A Mixed‐Methods Summative Evaluation With Health Professionals.” International Journal of Nursing Studies 174: 105308. 10.1016/j.ijnurstu.2025.105308. 41385997

[nop270908-bib-0042] Oesch, S. , L. Verweij , M. Riguzzi , T. Finch , and R. Naef . 2025. “Exploring Implementation Processes of a Multicomponent Family Support Intervention in Intensive Care Units (FICUS) Study: A Mixed‐Methods Process Evaluation.” Journal of Advanced Nursing 81, no. 11: 7245–7265. 10.1111/jan.16544. PMC12535341 39422155

[nop270908-bib-0043] Östlund, U. , B. Bäckström , V. Lindh , K. Sundin , and B. I. Saveman . 2015. “Nurses' Fidelity to Theory‐Based Core Components When Implementing Family Health Conversations ‐ a Qualitative Inquiry.” Scandinavian Journal of Caring Sciences 29, no. 3: 582–590. 10.1111/scs.12178. 25236840

[nop270908-bib-0044] Paez, A. , P. McCulloch , and D. Beard . 2024. “The CONSIDER Intervention Fidelity Framework for Complex Interventions in Healthcare: A Best‐Fit Framework Synthesis.” 10.1101/2024.08.29.24312797.

[nop270908-bib-0045] Páez, A. , D. Nunan , P. McCulloch , and D. Beard . 2025. “The Influence of Intervention Fidelity on Treatment Effect Estimates in Clinical Trials of Complex Interventions: A Metaepidemiological Study.” Journal of Clinical Epidemiology 177: 111610. 10.1016/j.jclinepi.2024.111610. 39528004

[nop270908-bib-0046] Proctor, E. , H. Silmere , R. Raghavan , et al. 2011. “Outcomes for Implementation Research: Conceptual Distinctions, Measurement Challenges, and Research Agenda.” Administration and Policy in Mental Health 38, no. 2: 65–76. 10.1007/s10488-010-0319-7. PMC3068522 20957426

[nop270908-bib-0047] Proctor, E. K. , A. C. Bunger , R. Lengnick‐Hall , et al. 2023. “Ten Years of Implementation Outcomes Research: A Scoping Review.” Implementation Science 18, no. 1: 31. 10.1186/s13012-023-01286-z. PMC10367273 37491242

[nop270908-bib-0048] Regmi, K. , and L. Jones . 2020. “A Systematic Review of the Factors ‐ Enablers and Barriers ‐ Affecting e‐Learning in Health Sciences Education.” BMC Medical Education 20, no. 1: 91. 10.1186/s12909-020-02007-6. PMC7106784 32228560

[nop270908-bib-0049] Rhoads, S. L. , T. A. Trikalinos , M. M. Levy , and T. Amass . 2022. “Intensive Care Based Interventions to Reduce Family Member Stress Disorders: A Systematic Review of the Literature.” Journal of Critical Care Medicine 8, no. 3: 145–155. 10.2478/jccm-2022-0014. PMC9396952 36062043

[nop270908-bib-0050] Richards, D. , and I. Hallberg . 2015. Complex Interventions in Health: An Overview of Research Methods. Routledge. 10.4324/9780203794982.

[nop270908-bib-0051] Richardson, C. , K. Wicking , N. Biedermann , and T. Langtree . 2023. “Coaching in Nursing: An Integrative Literature Review.” Nursing Open 10, no. 10: 6635–6649. 10.1002/nop2.1925. PMC10495732 37365717

[nop270908-bib-0052] Riguzzi, M. , M. M. Jeitziner , M. Rufer , et al. 2026. “The FICUS Cluster Randomized Controlled Trial of a Family Support Intervention in Adult Intensive Care Units: Mental Health and Family Functioning Outcomes.” Intensive Care Medicine 52, no. 4: 637–650. 10.1007/s00134-026-08425-2. PMC13221400 42149244

[nop270908-bib-0053] Saeid, Y. , M. M. Salaree , A. Ebadi , and S. T. Moradian . 2020. “Family Intensive Care Unit Syndrome: An Integrative Review.” Iranian Journal of Nursing and Midwifery Research 25, no. 5: 361–368. 10.4103/ijnmr.IJNMR_243_19. PMC7737832 33344205

[nop270908-bib-0054] SAMS . 2015. Research With Human Subjets Handbook. 2nd ed. Swiss Academic for Medical Sciences. https://www.samw.ch/en/Publications/Handbooks.html.

[nop270908-bib-0055] Schultes, M. T. , M. Aijaz , J. Klug , and D. L. Fixsen . 2021. “Competences for Implementation Science: What Trainees Need to Learn and Where They Learn It.” Advances in Health Sciences Education: Theory and Practice 26, no. 1: 19–35. 10.1007/s10459-020-09969-8. PMC7900055 32372393

[nop270908-bib-0056] Shajani, Z. , and D. Snell . 2023. Wright & Leahey's Nurses and Families: A Guide to Family Assessment and Intervention. 8th ed. F. A. Davis.

[nop270908-bib-0057] Shirasaki, K. , T. Hifumi , N. Nakanishi , et al. 2024. “Postintensive Care Syndrome Family: A Comprehensive Review.” Acute Medicine & Surgery 11, no. 1: e939. 10.1002/ams2.939. PMC10928249 38476451

[nop270908-bib-0058] Simpson, P. , and M. Tarrant . 2006. “Development of the Family Nursing Practice Scale.” Journal of Family Nursing 12, no. 4: 413–425. 10.1177/1074840706290806. 17099118

[nop270908-bib-0059] Skivington, K. , N. Craig , P. Craig , et al. 2024. “Introducing the Revised Framework for Developing and Evaluating Complex Interventions: A Challenge and a Resource for Nursing Research.” International Journal of Nursing Studies 154: 104748. 10.1016/j.ijnurstu.2024.104748. 38564983

[nop270908-bib-0060] Skivington, K. , L. Matthews , S. A. Simpson , et al. 2024. “A New Framework for Developing and Evaluating Complex Interventions: Update of Medical Research Council Guidance.” International Journal of Nursing Studies 154: 104705. 10.1016/j.ijnurstu.2024.104705. 38564982

[nop270908-bib-0061] Smith, A. C. , H. N. Ferguson , R. M. Russell , P. Savsani , and S. Wang . 2025. “Post‐Intensive Care Syndrome Family.” Critical Care Clinics 41, no. 1: 73–88. 10.1016/j.ccc.2024.08.008. PMC11573244 39547728

[nop270908-bib-0062] Walton, H. 2024. “Towards Comprehensive Fidelity Evaluations: Consideration of Enactment Measures in Quality Improvement Interventions.” BMJ Quality and Safety 33, no. 2: 78–81. 10.1136/bmjqs-2023-016592. 37714701

[nop270908-bib-0063] Wiltsey Stirman, S. , A. A. Baumann , and C. J. Miller . 2019. “The FRAME: An Expanded Framework for Reporting Adaptations and Modifications to Evidence‐Based Interventions.” Implementation Science 14, no. 1: 58. 10.1186/s13012-019-0898-y. PMC6554895 31171014

[nop270908-bib-0064] Xyrichis, A. , S. Fletcher , J. Philippou , S. Brearley , M. Terblanche , and A. M. Rafferty . 2021. “Interventions to Promote Family Member Involvement in Adult Critical Care Settings: A Systematic Review.” BMJ Open 11, no. 4: e042556. 10.1136/bmjopen-2020-042556. PMC8031009 33827833

[nop270908-bib-0065] Zvoch, K. 2012. “How Does Fidelity of Implementation Matter? Using Multilevel Models to Detect Relationships Between Participant Outcomes and the Delivery and Receipt of Treatment.” American Journal of Evaluation 33, no. 4: 547–565. 10.1177/1098214012452715.

