# Supplementary material for: Family Nurses' Experience With and Fidelity to a Family Support Intervention in Intensive Care Units: A Mixed‐Methods Study
Source: Nurs Open. 2026 Nov 13;13(11):e70908. doi: 10.1002/nop2.70908 (PMC13768588; doi:10.1002/nop2.70908)
Supplement: Supplementary file 1 [file NOP2-13-e70908-s001.docx]

Supplementary file S3. Interview Guide (condensed version)

Family nurses’ experience with and fidelity to a family support intervention in intensive care units: A mixed methods study

Running title: Fidelity results from the FICUS-trial

Authors: Lotte VERWEIJ PhD, RN^1,2^, Saskia OESCH PhD, RN^1,2*^, Marco RIGUZZI PhD^1,2^, Simone SUTTER MSc.^1,2^, Rahel NAEF PhD, RN^1,2^

^1^ University of Zurich, Faculty of Medicine, Implementation Science in Nursing, Plattenstrasse 10, 8032, Zurich Switzerland.

^2^ University Hospital Zurich, Center for Clinical Nursing Science, Rämistrasse 100, 80.91 Zurich Switzerland

*Current affiliation: Careum School of Health, Gloriastrasse 18a, 8006 Zurich, Switzerland

| **Phase** | **Content** |
| --- | --- |
| Welcome & introduction | Introduction of moderators; explanation of study aims, methodology, confidentiality, voluntary participation, and audio recording; communication rules; informed consent. Brief overview of the four discussion topics, with emphasis on experiences with the intervention rather than study procedures. |
| A. Role of the family nurse | Experiences with the family nurse role within the interprofessional team; perceived challenges and facilitators; impact of the role on team dynamics and collaboration. |
| B. Intervention delivery in the intensive care context | Experiences with delivering the family support intervention in daily intensive care practice; challenges related to acceptance, feasibility, and implementation; factors that supported successful delivery. |
| C. Implementation process | Perceived usefulness of implementation strategies (e.g., training, case conferences, coaching, implementation support); unmet needs and suggestions for improvement. |
| D. Perceived effects and impact | Perceived benefits of the family support intervention for nurses, teams, and families; unintended consequences; particularly effective or less effective intervention components; suggestions for adaptation or refinement. |
| E. Future sustainability | Perspectives on continuation of the intervention beyond the study period; perceived conditions and requirements for sustainability in the intensive care unit. |
| Closing | Final reflections, additional comments, thanks and closure. |
