# Supplementary material for: Family Nurses' Experience With and Fidelity to a Family Support Intervention in Intensive Care Units: A Mixed‐Methods Study
Source: Nurs Open. 2026 Nov 13;13(11):e70908. doi: 10.1002/nop2.70908 (PMC13768588; doi:10.1002/nop2.70908)
Supplement: Supplementary file 2 [file NOP2-13-e70908-s002.docx]

Supplementary file S2. Self-developed family support intervention self-rating and observation tool(1).

Family nurses’ experience with and fidelity to a family support intervention in intensive care units: A mixed methods study

Running title: Fidelity results from the FICUS-trial

Authors: Lotte VERWEIJ PhD, RN^1,2^, Saskia OESCH PhD, RN^1,2*^, Marco RIGUZZI PhD^1,2^, Simone SUTTER MSc.^1,2^, Rahel NAEF PhD, RN^1,2^

^1^ University of Zurich, Faculty of Medicine, Implementation Science in Nursing, Plattenstrasse 10, 8032, Zurich Switzerland.

^2^ University Hospital Zurich, Center for Clinical Nursing Science, Rämistrasse 100, 80.91 Zurich Switzerland

*Current affiliation: Careum School of Health, Gloriastrasse 18a, 8006 Zurich, Switzerland

FSI component 1. Engaging and liaising

| **Indicator** | **Operationalization** | **Self-rating** | **Observation** |
| --- | --- | --- | --- |
| **Core dimensions** |  |  |  |
| Relationship building | 1. I establish contact with the family as early as possible after admission. | x | x |
| Conducting check-in conversations | 2. I conduct check-in conversations according to the intervention manual. | x | x |
| Coordination activities | 3. I coordinate care and communication with the family. | x | x |
| **Activities** |  |  |  |
| Introduction | 4. I introduce myself personally and inform the family about my role as a family nurse (primary contact person). | x | x |
|  | 5. I identify who belongs to the family, including the primary family contact, the study participant, and the legal representative. | x | x |
| Establishing equality | 6. I regard the family as an equal partner. | x | x |
|  | 7. I acknowledge that the family brings strengths and resources. | x | x |
| Expressing appreciation | 8. I express appreciation for what the family has already accomplished and/or acknowledge their burden, uncertainty, and concerns. | x | x |
| Introducing the ICU | 9. I introduce the family to ICU routines and show them the facilities. | x | x |
|  | 10. I introduce the responsible interprofessional ICU team by name. | x | x |
| Ensuring continuity of care | 11. I maintain contact with the family through regular communication (in person, phone, email). | x |  |
| Interprofessional coordination | 12. I ensure that family care is coordinated and delivered consistently within the interprofessional team. | x | x |
| Referrals | 13. If needed, I initiate consultations and refer the family to additional professional support (e.g. social work, chaplaincy, psychology/psychiatry, clinical ethics, care team, general practitioner). | x | x |
| Transitions and follow-up care | 14. I support the family during transfer to another ward/ICU/institution. | x | x |
|  | 15. I support the family during the dying process. | x | x |
|  | 16. I ensure coordinated family care by planning family meetings at discharge/transfer/death and follow-up conversations. | x | x |

Legend: ICU= intensive care unit

FSI component 2. Supporting / family conversations

| **Indicator** | **Operationalization** | **Self-rating** | **Observation** |
| --- | --- | --- | --- |
| **Core dimensions** |  |  |  |
| Relationship building | 17. I build relationships with family members through family conversations. | x | x |
| Conducting family conversations | 18. I conduct family conversations according to the intervention manual. | x | x |
| **Activities** |  |  |  |
| Bringing family together | 19. I invite all relevant family members and enable their participation. |  |  |
| Enabling illness narrative | 20. I encourage the family to talk about their current situation. | x | x |
| Assessing family structure | 21. I get to know the family by jointly creating a genogram/ecomap/relationship diagram. | x | x |
|  | 22. Together with the family, I explore priorities, preferences, and support needs. | x | x |
| Identifying patterns | 23. Together with the family, I explore how they have coped with similar situations in the past. | x | x |
| Perceiving emotions | 24. I assess the wellbeing of each family member and the family as a whole. | x | x |
| Exploring illness understanding | 25. I assess the family’s knowledge and beliefs about the illness trajectory. | x | x |
| Enabling family presence | 26. I enable families to be with the patient and support family routines and rituals in the ICU. | x | x |
| Identifying resources | 27. I identify and make visible the family’s own and available resources. | x | x |
| Strengthening agency | 28. I strengthen families emotionally, cognitively, and behaviorally. | x | x |
| Strengthening relationships | 29. I connect family members, promote mutual understanding, and strengthen relationships. | x | x |
| Validating emotions | 30. I acknowledge and validate the family’s emotions and experiences. | x | x |
| Self-care | 31. I support families in self-management and self-care. | x | x |
| Additional support | 32. Together with the family, I assess whether additional support is needed. | x | x |
| Systemic questioning | 33. I use systemic, circular, and interventive questions. | x | x |
|  | 34. I encourage the family to elaborate and deepen their narrative. | x | x |
| Active listening | 35. I actively listen and express appreciation. | x | x |
|  | 36. I acknowledge similarities and differences within the family. | x | x |
| Counselling | 37. I counsel the family on coping with severe illness and its impact on daily life. | x | x |
|  | 38. If applicable, I counsel the family regarding dying, loss, and grief. | x | x |
| Education | 39. I educate the family as needed about illness and intensive care treatment. | x | x |
| Information | 40. I provide timely, consistent, and understandable information. | x | x |
| Action planning | 41. I explore what would help or relieve the family most at present. | x | x |
|  | 42. I develop an action plan together with the family. | x | x |
|  | 43. I evaluate with the family how helpful the agreed measures were. | x | x |
|  | 44. I clarify what I can concretely do for the family as a family nurse. | x | x |

Legend: ICU= intensive care unit

FSI component 3. Communicating / interprofessional family meetings

| **Indicator** | **Operationalization** | **Self-rating** | **Observation** |
| --- | --- | --- | --- |
| **Core dimensions** |  |  |  |
| Relationship building | 45. I build relationships with the family through interprofessional family meetings. | x | x |
| Co-moderation | 46. I conduct interprofessional family meetings according to the intervention manual. | x | x |
| Interprofessional collaboration | 47. I initiate and maintain appropriate, goal-oriented interprofessional collaboration. | x | x |
| **Activities** |  |  |  |
| Initiation and planning | 48. I initiate and plan interprofessional family meetings in coordination with the family and care teams. | x | x |
| Preparation | 49. Together with physicians and other professionals, I clarify or define a shared goal for the meeting. | x | x |
| Co-moderation | 50. I co-moderate interprofessional family meetings. | x | x |
|  | 51. I act as a mediator between family and team. | x | x |
| Documentation | 52. I document agreements, activities, and procedures in the patient record. | x | x |
| Exploring VALUE* | 53. We explore the family’s experiences, concerns, questions, and perspectives. | x | x |
|  | 54. We identify the issues that currently concern the family most. | x | x |
| Informing | 55. We ensure that the family’s key questions are addressed. | x | x |
| Shared decision-making | 56. We interact with the family as equal partners and respect their perspectives and preferences. | x | x |
|  | 57. We facilitate shared decision-making between the care team and the family. |  |  |
| Care planning | 58. We agree on next steps/action plans with the family. | x | x |
|  | 59. We schedule a follow-up family meeting. | x | x |
|  | 60. We inform the family that they can contact us at any time. | x | x |

Legend: *VALUE = Value, Acknowledge, Listen, Understand, Elicit questions, ICU= intensive care unit

Answer categories

|  | **Self-rating** | **Observation** |
| --- | --- | --- |
| Rating options | 1. Never succeeds 2. Rarely succeeds 3. Occasionally succeeds 4. Often succeeds 5. Always succeeds | 1. Performed 2. Partly performed 3. Not performed 0. Not observable or not applicable |

1.	Oesch S, Verweij L, Clack L, Finch T, Riguzzi M, Naef R. Implementation of a multicomponent family support intervention in adult intensive care units: study protocol for an embedded mixed-methods multiple case study (FICUS implementation study). BMJ Open. 2023;13(8):e074142.
