# Supplementary material for: Family Nurses' Experience With and Fidelity to a Family Support Intervention in Intensive Care Units: A Mixed‐Methods Study
Source: Nurs Open. 2026 Nov 13;13(11):e70908. doi: 10.1002/nop2.70908 (PMC13768588; doi:10.1002/nop2.70908)
Supplement: Supplementary file 3 [file NOP2-13-e70908-s003.docx]

Supplementary file S1. Good reporting of a Mixed Methods Study (GRAMMS)

Family nurses’ experience with and fidelity to a family support intervention in intensive care units: A mixed methods study

Running title: Fidelity results from the FICUS-trial

Authors: Lotte VERWEIJ PhD, RN^1,2^, Saskia OESCH PhD, RN^1,2*^, Marco RIGUZZI PhD^1,2^, Simone SUTTER MSc.^1,2^, Rahel NAEF PhD, RN^1,2^

^1^ University of Zurich, Faculty of Medicine, Implementation Science in Nursing, Plattenstrasse 10, 8032, Zurich Switzerland.

^2^ University Hospital Zurich, Center for Clinical Nursing Science, Rämistrasse 100, 80.91 Zurich Switzerland

*Current affiliation: Careum School of Health, Gloriastrasse 18a, 8006 Zurich, Switzerland

| **Guideline** | **Section** | **Page** |
| --- | --- | --- |
| Describe the justification for using a mixed methods approach to the research question | Introduction – aim  Methods - Design | 4-5 |
| Describe the design in terms of the purpose, priority, and sequence of methods | Methods – Design,  Setting and participants,  Data collection | 5-10 |
| Describe each method in terms of sampling, data collection and analysis | Methods- Data collection,  Data analysis | 7-12 |
| Describe where integration has occurred, how it has occurred and who has participated in it | Results, table 4  Discussion | 23-27 |
| Describe any limitation of one method associated with the present of the other method | Discussion - Strength and limitations | 28 |
| Describe any insights gained from mixing or integrating methods | Discussion | 24-29 |

O'Cathain A, Murphy E, Nicholl J. (2008). The quality of mixed methods studies in health services research. *Journal of Health Services Research & Policy*. 13: 92-98.
